# Laparoscopic *versus* open right hepatectomy for colorectal liver metastases after portal vein embolization: international multicentre study

**DOI:** 10.1093/bjs/znae181

**Published:** 2024-08-13

**Authors:** Emre Bozkurt, Jasper P Sijberden, Serena Langella, Federica Cipriani, Francesc Collado-Roura, Victoria Morrison-Jones, Burak Görgec, Gabriel Zozaya, Jacopo Lanari, Davit Aghayan, Celine De Meyere, David Fuks, Giuseppe Zimmiti, Benedetto Ielpo, Mikhail Efanov, Robert P Sutcliffe, Nadia Russolillo, Miquel Gomez-Artacho, Francesca Ratti, Mathieu D’Hondt, Bjørn Edwin, Umberto Cillo, Fernando Rotellar, Marc G Besselink, John N Primrose, Santi Lopez-Ben, Luca A Aldrighetti, Alessandro Ferrero, Mohammad Abu Hilal

**Affiliations:** Department of Surgery, Fondazione Poliambulanza Istituto Ospedaliero, Brescia, Italy; Department of Surgery, Koç University School of Medicine, Istanbul, Turkey; Department of Surgery, Fondazione Poliambulanza Istituto Ospedaliero, Brescia, Italy; Department of Surgery, Amsterdam UMC, location University of Amsterdam, Amsterdam, The Netherlands; Cancer Center Amsterdam, Amsterdam, The Netherlands; Department of General and Oncological Surgery, Umberto I Mauriziano Hospital, Turin, Italy; Hepatobiliary Surgery Division, IRCCS San Raffaele Hospital, Milan, Italy; Servei de Cirurgia General i Digestiva, Hospital Universitari Doctor Josep Trueta de Girona, Girona, Spain; Department of Surgery, University Hospital Southampton NHS Foundation Trust, Southampton, UK; Department of Surgery, Amsterdam UMC, location University of Amsterdam, Amsterdam, The Netherlands; Cancer Center Amsterdam, Amsterdam, The Netherlands; Department of Surgery, HPB and Liver Transplantation Unit, University Clinic, Universidad de Navarra, Institute of Health Research of Navarra (IdisNA), Pamplona, Spain; Department of Surgical, Oncological and Gastroenterological Sciences, General Surgery 2, Hepato-pancreato-biliary Surgery and Liver Transplantation, Padua University Hospital, Padua, Italy; The Intervention Centre and Department of HPB Surgery, Oslo University Hospital and Institute of Medicine, University of Oslo, Oslo, Norway; Department of Digestive and Hepatobiliary/Pancreatic Surgery, Groeninge Hospital, Kortrijk, Belgium; Department of Digestive, Oncologic and Metabolic Surgery, Institut Mutualiste Montsouris, Université Paris Descartes, Paris, France; Department of Surgery, Fondazione Poliambulanza Istituto Ospedaliero, Brescia, Italy; Hepatobiliary and Pancreatic Surgery Unit, Hospital del Mar, Hospital del Mar Medical Research Institute (IMIM), Universitat Pompeu Fabra, Barcelona, Spain; Department of Hepato-Pancreato-Biliary Surgery, Moscow Clinical Research Centre, Moscow, Russia; Liver Unit, Queen Elizabeth Hospital, Birmingham, UK; Department of General and Oncological Surgery, Umberto I Mauriziano Hospital, Turin, Italy; Servei de Cirurgia General i Digestiva, Hospital Universitari Doctor Josep Trueta de Girona, Girona, Spain; Hepatobiliary Surgery Division, IRCCS San Raffaele Hospital, Milan, Italy; Department of Digestive and Hepatobiliary/Pancreatic Surgery, Groeninge Hospital, Kortrijk, Belgium; The Intervention Centre and Department of HPB Surgery, Oslo University Hospital and Institute of Medicine, University of Oslo, Oslo, Norway; Department of Surgical, Oncological and Gastroenterological Sciences, General Surgery 2, Hepato-pancreato-biliary Surgery and Liver Transplantation, Padua University Hospital, Padua, Italy; Department of Surgery, HPB and Liver Transplantation Unit, University Clinic, Universidad de Navarra, Institute of Health Research of Navarra (IdisNA), Pamplona, Spain; Department of Surgery, Amsterdam UMC, location University of Amsterdam, Amsterdam, The Netherlands; Cancer Center Amsterdam, Amsterdam, The Netherlands; Department of Surgery, University Hospital Southampton NHS Foundation Trust, Southampton, UK; Servei de Cirurgia General i Digestiva, Hospital Universitari Doctor Josep Trueta de Girona, Girona, Spain; Hepatobiliary Surgery Division, IRCCS San Raffaele Hospital, Milan, Italy; Department of General and Oncological Surgery, Umberto I Mauriziano Hospital, Turin, Italy; Department of Surgery, Fondazione Poliambulanza Istituto Ospedaliero, Brescia, Italy; Department of Surgery, University Hospital Southampton NHS Foundation Trust, Southampton, UK

## Abstract

**Background:**

Laparoscopic liver surgery is increasingly used for more challenging procedures. The aim of this study was to assess the feasibility and oncological safety of laparoscopic right hepatectomy for colorectal liver metastases after portal vein embolization.

**Methods:**

This was an international retrospective multicentre study of patients with colorectal liver metastases who underwent open or laparoscopic right and extended right hepatectomy after portal vein embolization between 2004 and 2020. The perioperative and oncological outcomes for patients who underwent laparoscopic and open approaches were compared using propensity score matching.

**Results:**

Of 338 patients, 84 patients underwent a laparoscopic procedure and 254 patients underwent an open procedure. Patients in the laparoscopic group less often underwent extended right hepatectomy (18% *versus* 34.6% (*P* = 0.004)), procedures in the setting of a two-stage hepatectomy (42% *versus* 65% (*P* < 0.001)), and major concurrent procedures (4% *versus* 16.1% (*P* = 0.003)). After propensity score matching, 78 patients remained in each group. The laparoscopic approach was associated with longer operating and Pringle times (330 *versus* 258.5 min (*P* < 0.001) and 65 *versus* 30 min (*P* = 0.001) respectively) and a shorter length of stay (7 *versus* 8 days (*P* = 0.011)). The R0 resection rate was not different (71% for the laparoscopic approach *versus* 60% for the open approach (*P* = 0.230)). The median disease-free survival was 12 (95% c.i. 10 to 20) months for the laparoscopic approach *versus* 20 (95% c.i. 13 to 31) months for the open approach (*P* = 0.145). The median overall survival was 28 (95% c.i. 22 to 48) months for the laparoscopic approach *versus* 42 (95% c.i. 35 to 52) months for the open approach (*P* = 0.614).

**Conclusion:**

The advantages of a laparoscopic over an open approach for (extended) right hepatectomy for colorectal liver metastases after portal vein embolization are limited.

## Teaser

In this study, the safety and feasibility of laparoscopic right and extended right hepatectomy for colorectal liver metastases after portal vein embolization were evaluated. After propensity score matching, the only observed benefit in the laparoscopic group was a 1-day shorter hospital stay, with longer operating and Pringle times. The technical complexity of laparoscopy in this particular setting thus seems to limit its advantages over an open approach.

## Introduction

Metastases from colorectal cancer most commonly occur in the liver. Up to 20% of patients have liver metastases at the time of diagnosis and more than 30% develop metachronous metastases^1–3^. Due to the functional, oncological, and technical difficulties related to both disease extent and liver function status, only 10–20% of patients with colorectal liver metastases (CRLM) are eligible for treatment with curative intent^4^. When major liver resection is needed to clear the liver of disease, the main limiting factor is an insufficient future liver remnant (FLR). Nowadays, there are several methods to increase the volume and function of the FLR. Portal vein embolization (PVE) is often preferred over other FLR modulation techniques. This is likely related to its periprocedural safety and the longer hypertrophy time, which allows for the identification of aggressive disease indicated by tumour progression before the subsequent hepatectomy^5–7^.

Laparoscopic liver surgery has increasingly been adopted. A plethora of studies have demonstrated the benefits of laparoscopic liver surgery over open liver surgery in selected patients, in terms of reduced length of stay, time to functional recovery, and lower (liver-specific) morbidity rates^8–10^. Oncological outcomes of laparoscopic liver surgery have been shown to be at least non-inferior to the traditional open approach^11,12^. With the advancement of surgical techniques and growing experience in laparoscopic liver surgery, the laparoscopic approach is adopted for more challenging procedures^11,13–16^. Several studies have now shown that, in experienced hands, the laparoscopic approach is also associated with improved short-term outcomes in the setting of major hepatectomies^17–20^. Preoperative FLR modulation can however further increase the technical difficulty of major hepatectomies, due to the anatomical changes and perivascular inflammation it induces. Evidence for laparoscopic surgery after PVE is scarce^21–26^. The aim of this study was to compare the feasibility and oncological safety of laparoscopic and open right and extended right hepatectomy for CRLM after PVE.

## Methods

This was a retrospective multicentre study of consecutive adult patients with CRLM who underwent open or laparoscopic right and extended right hepatectomy after PVE between 2004 and 2020. An international multicentre database included data from 17 tertiary referral hepatobiliary centres was used to perform this study. The medical ethics committee of Brescia approved the study protocol (protocol number NP5329). Informed consent was not considered necessary due to the its retrospective nature and use of pseudonymized data. This study is reported in accordance with strengthening the reporting of observational studies in epidemiology (STROBE) statement^27^.

### Interventions and surgical technique

PVE was carried out using a transhepatic approach, with patients under sedation or local anaesthesia, and selective embolization was performed after portal venography. After an adequate waiting time, the FLR volume was evaluated using CT. When the FLR was deemed sufficient, the decision was made to proceed to surgery. Although some variability between centres probably existed, laparoscopic resections were generally performed using the following technique. A total of five trocars (one 5 mm to the subxiphoidal, one to the 2 cm above the midpoint between the right midclavicular line and the umbilicus, one 10 mm to the midclavicular line at the level of the umbilicus, one 5 mm to the anterior axillary line at the level of the umbilicus, and one 10 mm to the left midclavicular line at the level of the umbilicus) were placed. Intraoperative ultrasonography was used to identify the number of lesions and the size of the lesions, as well as their relationship with major vascular structures. The liver was fully mobilized by dissecting the falciform and coronary ligament. The right lobe was elevated and venous branches to the retrohepatic vena cava were clipped and dissected. The Makuuchi ligament was dissected and divided using a stapler and the right hepatic vein was slinged. After the complete mobilization of the liver, the hepatoduodenal ligament was dissected and the right hepatic artery and the right portal vein were slinged. At the discretion of the operating surgeon, an intermittent Pringle manoeuvre was used by encircling the hepatoduodenal ligament. After clamping the right liver inflow, the left hemi-liver was controlled using Doppler ultrasonography for venous and arterial flow. Indocyanine green was selectively used to confirm the parenchymal ischaemia demarcation during this phase. Generally, parenchymal transection was performed using an ultrasonic dissector or a bipolar vessel sealer for the superficial part of the liver and an ultrasonic aspirator for the deep parenchyma. Vascular and biliary structures were managed with sealing device or clips based on diameter. When a safe and sufficient transection area was obtained (which could be challenging due to embolic material), the right hepatic duct, the right hepatic vein, and the right portal vein were dissected and usually transected using a stapler.

### Definitions and outcomes

The procedure types were defined according to the Brisbane 2000 nomenclature^28^. A resection of segment five to eight was defined as a right hepatectomy and a resection of segment four to eight was defined as an extended right hepatectomy. Concurrent (non-cholecystectomy) procedures, such as pancreatic, gastric, colorectal, or diaphragmatic resections and biliary or vascular reconstructions, were defined as major concurrent procedures. Two-stage hepatectomy was defined as FLR clearance followed by FLR modulation and surgical removal of the contralateral liver lobe as a second stage^29^. Intraoperative incidents were defined and graded using the Oslo intraoperative unfavourable events grading system and postoperative morbidity was defined and graded using the Clavien–Dindo classification and is reported as overall and severe (Clavien–Dindo greater than or equal to grade III). Bile leak and liver failure were defined using the definition of the International Study Group of Liver Surgery^30,31^. Bile leak greater than or equal to grade A and liver failure greater than or equal to grade A were reported. Postoperative morbidity was evaluated within the first 30 days postoperatively and postoperative mortality constituted the 90-day or in-hospital mortality. The resection margin was considered radical (R0) when microscopically greater than 1 mm. Disease-free survival (DFS) and overall survival (OS) were defined as the interval (in months) between the date of surgery and the date when there was clinical evidence of disease recurrence or the date of death, respectively.

### Statistical analysis

Patients were stratified into two groups, based on surgical approach. The perioperative and oncological outcomes for both groups were compared before and after propensity score matching (PSM). A subgroup analysis, in which the converted laparoscopic procedures were excluded, was performed using PSM. A sensitivity analysis was conducted to assess the outcomes in high-volume minimally invasive liver surgical centres, with centres performing greater than or equal to 50 laparoscopic resections per year being defined as high-volume centres^32^.

Continuous data, not normally distributed, are reported as the median (interquartile range (i.q.r.)) and comparisons between groups were performed using the Mann–Whitney *U* test. Normality was assessed by visually inspecting histograms and Q-Q plots. Categorical data are reported as *n* (%) and comparisons between groups were performed using a chi-squared test or Fisher’s exact test, when appropriate. Single imputation was used to impute missing data, which were present in a ‘missing at random’ pattern (*Fig. S1*). The outcome data were not imputed. Propensity scores were calculated using a multivariable logistic regression model. Variables that might influence treatment allocation were entered as covariates in this model and included age, sex, ASA grade, history of previous hepatic surgery, number of lesions, size of the largest lesion, procedure type (right hepatectomy or extended right hepatectomy), one- or two-stage procedure, and major concurrent procedures. Patients who underwent laparoscopic liver surgery were matched to their ‘nearest-neighbour’ who underwent open liver surgery in a 1 : 1 ratio without replacement, using a small caliper width. Standardized differences were used to evaluate the balance after PSM. A standardized difference less than 0.1 was considered to indicate optimal balance. After PSM, categorical data were compared using McNemar’s test or marginal homogeneity as appropriate. Continuous data were compared using the Wilcoxon signed rank test. Discrete variables were entered in their original form in the PSM logistic regression model, except for the variables number of lesions (dichotomized to single lesion *versus* multiple lesions) and size of the largest lesion (dichotomized to less than 51 mm or greater than or equal to 51 mm). DFS and OS were assessed using the Kaplan–Meier method combined with a stratified log rank test (stratification on the propensity score). All analyses were performed using SPSS^®^ (IBM, Armonk, NY, USA; Statistics version 29.0) and R for Mac OS X version 4.2.1. Single imputation was performed using SPSS^®^ and PSM was performed using the MatchIt package in R^33^. A two-tailed *P* < 0.050 was considered statistically significant.

## Results

**Fig. 1:**
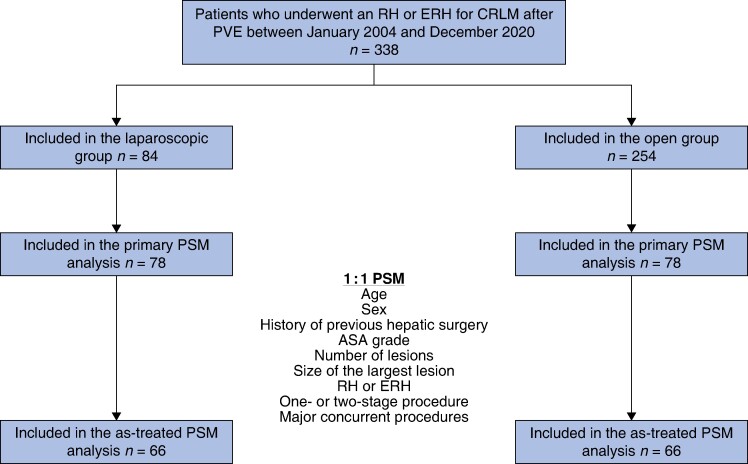
Study flow chart RH, right hepatectomy; ERH, extended right hepatectomy; CRLM, colorectal liver metastases; PVE, portal vein embolization; PSM, propensity score matching.

Overall, 338 patients were included, of whom 254 underwent open liver resection and 84 underwent laparoscopic liver resection. See *Fig. 1*. The use of the laparoscopic approach increased over time (*Table S1*).

### Characteristics and perioperative outcomes before propensity score matching

Patients in the laparoscopic group less often had a history of previous hepatic surgery and were more often treated with neoadjuvant chemotherapy (*Table 1*). They less often underwent extended right hepatectomy, procedures in the setting of a two-stage hepatectomy, and major concurrent procedures (*Table S2*). Patients who underwent laparoscopic surgery generally had less extensive disease, compared with patients who underwent open surgery, indicated by fewer and smaller lesions. The time interval from PVE to the definitive resection did not differ between the groups.

**Table 1:** Baseline, procedural, and disease characteristics of patients who underwent right and extended right hepatectomy for colorectal liver metastases after portal vein embolization stratified by surgical approach, before and after propensity score matching

	Before PSM		*P*	After PSM		Standardized difference	*P*
	Laparoscopic (*n* = 84)	Open (*n* = 254)		Laparoscopic (*n* = 78)	Open (*n* = 78)		
**Baseline characteristics**							
Age (years), median (i.q.r.)	65.5 (60.2–72.3)	63 (56–69)	0.007*	66 (60.5–72)	67 (60.3–71)	0.01	0.673
Male	61 (73)	171 (67.3)	0.364	55 (71)	55 (70.5)	0	1.000
BMI (kg/m^2^), median (i.q.r.)	25.3 (23.1–28.6)	25.1 (23–28)	0.855	25.3 (23.2–28.5)	24.7 (23–28.3)	0.02	0.682
ASA ≥grade III	41 (49)	114 (44.9)	0.531	38 (49)	40 (51)	0.05	0.880
Neoadjuvant chemotherapy	72 (86)	165 (65)	<0.001*	66 (85)	55 (71)	0.34	0.054
History of previous abdominal surgery	–	–	–	–	–	–	–
Extrahepatic	45 (54)	128 (50.4)	0.613	42 (54)	34 (44)	0.21	0.280
Hepatic	30 (36)	165 (65)	<0.001*	30 (39)	29 (37)	0.03	1.000
**Procedural characteristics**							
Time between PVE and resection (days), median (i.q.r.)	42.5 (34–59.5)	42 (31–60)	0.434	42 (34–57)	40 (30–54.5)	0.16	0.304
Extent of resection	–	–	0.004*	–	–	0.03	1.000
Right hepatectomy	69 (82)	166 (65.4)	–	63 (81)	62 (80)	–	–
Extended right hepatectomy	15 (18)	88 (34.6)	–	15 (19)	16 (21)	–	–
Part of two-stage hepatectomy	35 (42)	165 (65)	<0.001*	34 (44)	34 (44)	0	1.000
Major concurrent procedures	3 (4)	41 (16.1)	0.003*	3 (4)	4 (5)	0.06	1.000
**Disease characteristics**							
Bilobar distribution†	27 (32.1)	102 (40.2)	0.190	27 (35)	24 (31)	0.08	0.700
Number of lesions	4 (2–6)	5 (3–9)	0.022*	4 (2–6)	4 (2.3–7)	0.08	0.778
Size of the largest lesion (mm), median (i.q.r.)	33.5 (20–50)	40 (24.3–60)	0.035*	35 (21.8–53.8)	40 (22.5–55)	0.04	0.424

Values are *n* (%) unless otherwise indicated. *Statistically significant. †At the second stage in the case of a two-stage hepatectomy. PSM, propensity score matching; i.q.r., interquartile range; PVE, portal vein embolization.

A total of 11 laparoscopic procedures (13%) were converted to open procedures (*Table 2*); 5 conversions were performed due to bleeding, 4 conversions were performed due to technical difficulties, 1 conversion was performed to achieve oncological safety, and 1 conversion was performed directly after mobilization of the liver. Operating and Pringle times were longer in the laparoscopic group. The laparoscopic approach was associated with a lower bile leak rate and a shorter length of stay.

**Table 2:** Perioperative outcomes stratified by surgical approach, before and after propensity score matching

	Before PSM		*P*	After PSM		*P*
	Laparoscopic (*n* = 84)	Open (*n* = 254)		Laparoscopic (*n* = 78)	Open (*n* = 78)	
**Intraoperative outcomes**						
Operating time (min), median (i.q.r.)	329.5 (264.8–404.3)	286 (223.5–358)	0.002*	330 (268.3–420)	258.5 (212.5–313.8)	<0.001*
Estimated blood loss (ml), median (i.q.r.)	500 (236–950)	555 (292.5–967.3)	0.651	500 (227–940)	560 (295–1088)	0.845
Intraoperative PRBC transfusion	18 (24)	57 (27.1)	0.557	18 (25)	12 (19)	0.404
Number of PRBC transfused†, median (i.q.r.)	2 (2–2.5)	2 (2–3)	0.931	2 (2–2.5)	2 (1, 3.5)	0.382
Pringle manoeuvre	58 (69)	127 (59.3)	0.120	54 (69)	44 (63)	0.391
Total Pringle time when used (min), median (i.q.r.)	62.5 (31.5–93)	36.5 (24.8–59.3)	0.001*	65 (31.5–93.8)	30 (20–50)	0.001*
Intraoperative unfavourable incidents	–	–	0.074	–	–	0.095
Grade I	8 (11)	25 (17.6)	–	8 (12)	8 (16)	–
Grade II	4 (5)	3 (2.1)	–	4 (6)	1 (2)	–
Grade III	2 (3)	0	–	2 (3)	0	–
Conversion	11 (13)	–	–	11 (14)	–	–
Bleeding	5 (19)	–	–	5 (21)	–	–
Technical difficulty	4 (15)	–	–	4 (17)	–	–
Oncological safety	1 (4)	–	–	1 (4)	–	–
Planned after mobilization	1 (4)	–	–	1 (4)	–	–
**Postoperative outcomes**						
Overall morbidity	37 (44)	124 (53)	0.160	34 (44)	36 (50)	0.596
Bile leak ≥grade A	4 (5)	34 (15.2)	0.014*	4 (5)	7 (10)	0.547
Liver failure ≥grade A	4 (5)	18 (7.7)	0.364	4 (5)	4 (6)	1.000
Severe morbidity	18 (21)	44 (19)	0.638	17 (22)	13 (18)	0.689
Length of stay (days), median (i.q.r.)	6 (4–9)	9 (7–16)	<0.001*	7 (4–9)	8 (6–16)	0.011*
Readmission	7 (10)	14 (12.1)	0.576	6 (9)	6 (12)	1.000
Radical resection margin (R0)	60 (72)	189 (77.5)	0.340	54 (70)	58 (77)	0.391
Ninety-day or in-hospital mortality	4 (5)	12 (4.7)	0.989	4 (5)	4 (5)	1.000

Values are *n* (%) unless otherwise indicated. *Statistically significant. †For patients who received a transfusion. PSM, propensity score matching; i.q.r., interquartile range; PRBC, packed red blood cells.

### Characteristics and perioperative outcomes after propensity score matching

After PSM, 78 patients remained in each group (*Table 1*). Operating and Pringle times were longer in the laparoscopic group. Other intraoperative outcomes did not differ between the groups. The length of stay was 1 day shorter in the laparoscopic group. Other postoperative outcomes did not differ between the groups.

### Survival

**Fig. 2:**
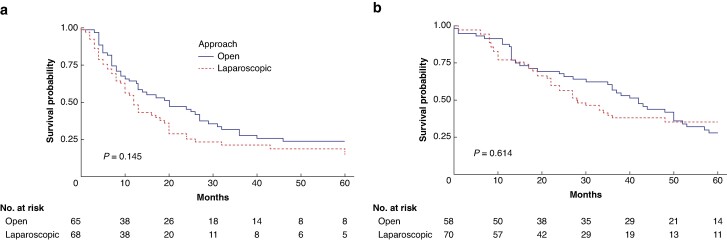
Survival of the propensity score matching cohort **a** Disease-free survival. **b** Overall survival.

Data regarding DFS and OS were available for 85% and 82% of the matched patients respectively. The median DFS (*Fig. 2a*) was 12 (95% c.i. 10 to 20) months for the laparoscopic approach versus 20 (95% c.i. 13 to 31) months for the open approach (*P* = 0.145) and the median OS (*Fig. 2b*) was 28 (95% c.i. 22 to 48) months for the laparoscopic approach versus 42 (95% c.i. 35 to 52) months for the open approach (*P* = 0.614).

### Characteristics and perioperative outcomes of the converted procedures and as-treated analysis

Patients who underwent converted procedures more often had a history of previous hepatic surgery (63% *versus* 32% (*P* = 0.038)) and more often underwent major concurrent procedures (18% *versus* 1% (*P* = 0.005)) compared with patients who underwent non-converted laparoscopic procedures (*Table S3*). The perioperative outcomes of the procedures that were converted, compared with procedures that were performed fully laparoscopically, are shown in *Table S4*. Converted procedures were associated with more blood loss, a higher transfusion rate, and higher overall and severe morbidity rates. Of 11 patients in the converted group, 2 patients (18%) died within the first 90 days after surgery, compared with 2 of 73 patients (3%) in the non-converted group (*P* = 0.025). The median length of stay was longer in the converted group.

After excluding the converted procedures, 66 of 67 remaining patients who underwent laparoscopic surgery were matched with 66 patients who underwent open surgery. The covariates were well balanced, but some imbalance remained for the covariates age (standardized difference 0.15), major concurrent procedures (standardized difference 0.18), and number of lesions (standardized difference 0.26). See *Table S5*. The length of stay was shorter for patients who underwent laparoscopic liver surgery without conversion. No other differences were observed. See *Table 3*.

**Table 3:** Perioperative outcomes after propensity score matching, excluding converted procedures

	Laparoscopic (*n* = 66)	Open (*n* = 66)	*P*
**Intraoperative outcomes**			
Operating time (min), median (i.q.r.)	300 (255–390)	252 (212.5–313.3)	0.015*
Estimated blood loss (ml), median (i.q.r.)	400 (200–700)	525 (265–805)	0.436
Intraoperative PRBC transfusion	9 (15)	7 (14)	0.773
Number of PRBC transfused†, median (i.q.r.)	2 (2–2)	2 (1–2)	0.832
Pringle manoeuvre	46 (70)	36 (59)	0.176
Total Pringle time when used (min), median (i.q.r.)	65 (32–93.5)	30 (23–45)	<0.001*
Intraoperative unfavourable incidents	–	–	0.257
Grade I	4 (7)	5 (12)	–
Grade II	0	0	–
Grade III	0	0	–
**Postoperative outcomes**			
Overall morbidity	23 (35)	26 (42)	0.596
Bile leak ≥grade A	3 (5)	4 (7)	1.000
Liver failure ≥grade A	3 (5)	3 (5)	1.000
Severe morbidity	9 (14)	8 (13)	1.000
Length of stay (days), median (i.q.r.)	6 (4–8)	7.8 (6–11.8)	0.004*
Readmission	6 (11)	5 (12)	1.000
Radical resection margin (R0)	49 (75)	56 (85)	0.286
Ninety-day or in-hospital mortality	1 (2)	5 (8)	0.221

Values are *n* (%) unless otherwise indicated. *Statistically significant. †For patients who received a transfusion. i.q.r., interquartile range; PRBC, packed red blood cells.

### Sensitivity analysis

The sensitivity analysis in high-volume centres generally yielded comparable results to the primary analysis. See *Tables S6 and S7*. The laparoscopic approach had a more pronounced benefit in terms of length of stay in these centres, with a median length of stay of 6 (i.q.r. 4–9) *versus* 8 (i.q.r. 7–14) days (*P* < 0.001).

## Discussion

Laparoscopic right and extended right hepatectomies for CRLM after PVE can be performed safely and effectively by experienced surgeons working in specialized centres. However, the advantages over the open approach in this setting are very limited. In this technically complex setting, most patients underwent open surgery. Patients were probably carefully selected for the laparoscopic approach, indicated by the fact that they had less extensive disease. Differences between the groups included longer operating and Pringle times, but only a 1-day shorter length of stay, for patients who underwent laparoscopic hepatectomy. Other outcomes, including R0 rates, DFS, and OS, did not differ between the groups.

A system for scoring the complexity of laparoscopic liver resections has been formulated. A positive correlation was observed between the difficulty index and prolonged operating time^34^. In this study, the operating and Pringle times were longer in the laparoscopic group, highlighting the technical complexity of these procedures. Laparoscopic liver resections have generally been associated with less blood loss^35,36^. However, in the context of major hepatectomy, the laparoscopic approach has been associated with comparable amounts of blood loss to open surgery, thereby losing this advantage^12,17–19^. This study had concordant results. Even when the converted cases were excluded, intraoperative blood loss did not differ between the two groups. Overall morbidity, severe morbidity, and liver-specific complications did not differ between the groups either. Observed liver failure rates were slightly lower than generally reported in the literature, possibly due to the selection of patients diagnosed with CRLM only^37^.

These findings support the importance of expanding indications for laparoscopic liver surgery only after completing the learning curve and employing proper patient selection. A history of previous hepatic surgery and the need for major concurrent procedures demand specific consideration during patient selection. In this study, the conversion rate was 13%, concordant with rates reported for laparoscopic major liver resections in general of between 0% and 11%^38^. It is well known that emergency conversions are associated with poor outcomes^39^.

Several studies have provided insights into the impact of centre volume on the outcomes of minimally invasive liver surgery^32,40^. The proficiency of the surgeon constitutes another pivotal determinant impacting postoperative results^41–43^. Notably, the surgeons participating in this study routinely perform more than 20 minimally invasive hepatectomies on an annual basis.

The most frequently mentioned benefit of minimally invasive surgery is its ability to accelerate functional recovery, indicated by a shorter hospital stay^8,44^. The clinical relevance of a 1-day reduction in hospital stay in the laparoscopic group, and its associated cost-effectiveness, is a matter of debate^44,45^.

This study has several limitations. These include the relatively small sample size and retrospective design, without standardization of perioperative care and surgical techniques. Data on FLR changes after PVE, which could be a crucial factor in the occurrence of liver failure, were not available. It is also important to note that the laparoscopic approach was generally used for more straightforward cases. PSM might not have been completely able to adjust for confounding by indication. Concludingly, the findings of this study indicate that laparoscopic right hepatectomy for CRLM after PVE can be performed safely and effectively by experienced surgeons working in specialized centres. However, it should be acknowledged that the advantages over the open approach in this setting are very limited when compared to those seen in other less complex resections.

## Supplementary Material

znae181_Supplementary_Data

## Data Availability

The data that support the findings of this study are available from the corresponding author, M.A.H., upon reasonable request. The data are not publicly available as this could compromise the privacy of research participants.
