# Supplementary material for: Laparoscopic versus open right hepatectomy for colorectal liver metastases after portal vein embolization: international multicentre study
Source: Br J Surg. 2024 Aug 13;111(8):znae181. doi: 10.1093/bjs/znae181 (PMC11319932; doi:10.1093/bjs/znae181)
Supplement: znae181_Supplementary_Data [file znae181_supplementary_data.docx]

**Supplementary Materials – Index**

| **Supplementary table 1.** Patients enrolled that underwent laparoscopic right or extended right hepatectomies per year | *pag. 2* |
| --- | --- |
| **Supplementary table 2.** Major (non-cholecystectomy) concurrent procedures per treatment group | *pag. 2* |
| **Supplementary table 3.** Characteristics of non-converted versus converted cases  **Supplementary table 4.** Perioperative outcomes of non-converted versus converted procedures | *pag. 3*  *pag. 4* |
| **Supplementary table 5.** Baseline, procedural and disease characteristics stratified by treatment group, after propensity score matching**,** excluding converted cases | *pag. 5* |
| **Supplementary table 6.** Baseline, procedural and disease characteristics stratified by treatment group, after propensity score matching, only high-volume centres | *pag. 6* |
| **Supplementary table 7.** Perioperative outcomes stratified by treatment group, after propensity score matching, only high-volume centres | *pag. 7* |
| **Supplementary figure 1.** Overview of missing data | *pag. 8* |
|  |  |

**Supplementary Figures and Tables**

| **Supplementary table 1.** Patients enrolled that underwent laparoscopic right or extended right hepatectomies per year | | | | | | | | | | | | | | |
| --- | --- | --- | --- | --- | --- | --- | --- | --- | --- | --- | --- | --- | --- | --- |
| 2004 | 2005 | 2006 | 2008 | 2009 | 2010 | 2012 | 2013 | 2014 | 2015 | 2016 | 2017 | 2018 | 2019 | 2020 |
| 1 | 1 | 1 | 1 | 3 | 1 | 4 | 1 | 5 | 14 | 18 | 10 | 14 | 6 | 4 |

| **Supplementary table 2.** Major (non-cholecystectomy) concurrent procedures by treatment group | | |  |
| --- | --- | --- | --- |
|  |  |  |  |
| *Type of procedure* | Laparoscopic | Open |  |
|  | n = 3 | n = 41 |  |
| Bile duct resection | 0 | 2 |  |
| Bile duct resection and hepaticojejunostomy | 0 | 3 |  |
| Biliary reconstruction | 0 | 3 |  |
| Biliary reconstruction, diaphragmatic resection | 0 | 1 |  |
| Colorectal resection | 2 | 7 |  |
| Diaphragmatic resection | 1 | 8 |  |
| Diaphragmatic resection, gastric resection | 0 | 1 |  |
| Hepaticojejunostomy | 0 | 2 |  |
| Hepaticojejunostomy, portal thrombectomy | 0 | 1 |  |
| Hepaticojejunostomy, portal vein resection | 0 | 1 |  |
| Porta ligation | 0 | 1 |  |
| Resection inferior caval vein | 0 | 4 |  |
| Splenectomy | 0 | 1 |  |
| Splenic artery ligation | 0 | 1 |  |
| Venotomy, trombectomy | 0 | 1 |  |
| Missing | 0 | 4 |  |

| **Supplementary table 3.** Characteristics of non-converted versus converted cases | | | |  |
| --- | --- | --- | --- | --- |
|  |  |  |  |  |
|  | Non-converted | Converted | P |  |
|  | n = 73 | n = 11 |  |  |
| *Baseline characteristics* | | | |  |
| Age, years | 65 [58, 72] | 68 [60.7, 74.5] | 0.582 |  |
| Gender, male | 53 (72.6) | 8 (72.7) | 0.993 |  |
| BMI | 24.7 [22.6, 28.3] | 26 [25.3, 29.1] | 0.168 |  |
| ASA-score 3&4 | 34 (46.6) | 7 (63.6) | 0.291 |  |
| Neoadjuvant chemotherapy | 61 (83.6) | 11 (100) | 0.146 |  |
| Previous abdominal surgery |  |  |  |  |
| Extrahepatic | 39 (53.4) | 6 (54.5) | 0.945 |  |
| Hepatic | 23 (31.5) | 7 (63.6) | 0.038 |  |
| *Procedural characteristics* | | | |  |
| Time interval PVE to resection, days | 42 [34, 56] | 59.5 [29.5, 84.3] | 0.368 |  |
| Extent of resection |  |  | 0.976 |  |
| Right hepatectomy | 60 (82.2) | 9 (81.8) |  |  |
| Extended right hepatectomy | 13 (17.8) | 2 (18.2) |  |  |
| Part of two-stage hepatectomy | 28 (38.4) | 7 (63.6) | 0.113 |  |
| Major concurrent procedure(s) | 1 (1.4) | 2 (18.2) | 0.005 |  |
| *Disease characteristics* | | | |  |
| Bilobar distribution* | 23 (31.5) | 4 (36.4) | 0.748 |  |
| Number of lesions | 4 [2, 6] | 6 [3.5, 12] | 0.066 |  |
| Size largest lesion, millimetres | 33 [20, 45] | 55 [20.5, 64] | 0.385 |  |
| Values are expressed in counts (percentages) or in median (IQR).  Abbreviations: BMI, body mass index; ASA, American Society of Anaesthesiologists; PVE, portal vein embolization.  *At the second-stage in case of a two-stage hepatectomy | | | |  |

| **Supplementary table 4.** Perioperative outcomes of non-converted versus converted procedures | | | |
| --- | --- | --- | --- |
|  | Non-converted | Converted | P |
|  | n = 73 | n = 11 |  |
| *Intraoperative outcomes* | | | |
| Operative time (minutes) | 315 [257.5, 394.5] | 360 [322, 485] | 0.100 |
| Estimated blood loss (millilitres) | 450 [218, 770] | 1450 [550, 3480.5] | 0.027 |
| Intraoperative PRBC transfusion | 11 (16.7) | 7 (70) | <0.001 |
| Number of PRBC transfused** | 2 [2, 2] | 3 [1.25, 5.5] | 0.480 |
| Pringle manoeuvre | 51 (69.9) | 58 (69) | 0.677 |
| Total Pringle time when used (minutes) | 65 [33, 93.5] | 40 [19, 76] | 0.340 |
| Intraoperative unfavourable incidents |  |  | <0.001 |
| Grade I | 5 (7.9) | 3 (27.3) |  |
| Grade II | 0 | 4 (36.4) |  |
| Grade III | 0 | 2 (18.2) |  |
| *Postoperative outcomes* | | | |
| Overall morbidity | 28 (38.4) | 9 (81.8) | 0.002 |
| Bile leak (≥ grade A) | 4 (5.6) | 0 | 0.423 |
| Liver failure (≥ grade A) | 3 (4.1) | 1 (9.1) | 0.470 |
| Severe morbidity | 12 (16.4) | 6 (54.5) | 0.007 |
| Length of stay (days) | 6 [4, 9] | 9 [9, 20.5] | 0.002 |
| Readmission | 6 (9.5) | 1 (9.1) | 0.964 |
| Radical resection margin (R0) | 53 (73.6) | 7 (63.3) | 0.491 |
| 90-day or in-hospital mortality | 2 (2.7) | 2 (18.2) | 0.025 |
| Values are expressed in counts (percentages) or in median (IQR).  Abbreviations: PRBC, packed red blood cell.  *Converted procedures excluded  **For patients that received a transfusion | | | |

| **Supplementary table 5.** Baseline, procedural and disease characteristics stratified by treatment group, after propensity score matching**, excluding converted cases** | | | | |  |
| --- | --- | --- | --- | --- | --- |
|  |  |  |  |  |  |
|  | Overall | | SD | P |  |
|  |  |  |  |  |  |
|  | Laparoscopic | Open |  |  |  |
|  | n = 66 | n = 66 |  |  |  |
| *Baseline characteristics* | | | | |  |
| Age, years | 66 [61.1, 73.8] | 66.4 [58.9, 71] | 0.15 | 0.041 |  |
| Gender, male | 46 (69.7) | 47 (71.2) | 0.03 | 1 |  |
| BMI | 24.8 [22.1, 28.5] | 25.7 [23.8, 28.5] | 0.24 | 0.225 |  |
| ASA-score 3&4 | 30 (45.5) | 31 (47.0) | 0.03 | 1 |  |
| Neoadjuvant chemotherapy | 54 (81.8) | 46 (69.7) | 0.29 | 0.153 |  |
| Previous abdominal surgery |  |  |  |  |  |
| Extrahepatic | 35 (53.0) | 31 (47.0) | 0.12 | 0.571 |  |
| Hepatic | 23 (34.8) | 25 (37.9) | 0.06 | 0.752 |  |
| *Procedural characteristics* | | | | |  |
| Time interval PVE to resection, days | 42 [34, 56] | 39.5 [30, 55] | 0.185 | 0.627 |  |
| Extent of resection |  |  | 0 | 1 |  |
| Right hepatectomy | 53 (80.3) | 53 (80.3) |  |  |  |
| Extended right hepatectomy | 13 (19.7) | 13 (19.7) |  |  |  |
| Part of two-stage hepatectomy | 26 (39.4) | 28 (42.4) | 0.06 | 0.803 |  |
| Major concurrent procedure(s) | 1 (1.5) | 0 | 0.18 | 1 |  |
| *Disease characteristics* | | | | |  |
| Bilobar distribution* | 21 (31.8) | 21 (31.8) | 0 | 1 |  |
| Number of lesions | 4 [2, 6] | 4 [2, 7] | 0.26 | 0.307 |  |
| Size largest lesion, millimetres | 32.5 [20, 45] | 31.4 [15.8, 47.8] | 0.07 | 0.774 |  |
| Values are expressed in counts (percentages) or in median (IQR).  Abbreviations: SD, standardized difference; BMI, body mass index; ASA, American Society of Anaesthesiologists; PVE, portal vein embolization.  *At the second-stage in case of a two-stage hepatectomy | | | | |  |

| **Supplementary table 6.** Baseline, procedural and disease characteristics stratified by the treatment group, after propensity score matching**, only high-volume centres** | | | | |  |
| --- | --- | --- | --- | --- | --- |
|  |  |  |  |  |  |
|  | Overall | | SD | P |  |
|  |  |  |  |  |  |
|  | Laparoscopic | Open |  |  |  |
|  | n = 59 | n = 59 |  |  |  |
| *Baseline characteristics* | | | | |  |
| Age, years | 64 [59.5, 71.5] | 67 [57.5, 71] | 0.06 | 0.724 |  |
| Gender, male | 25.3 [23.1, 28.6] | 25 [22.7, 27.6] | 0 | 1 |  |
| BMI | 44 (74.6) | 43 (72.9) | 0.04 | 0.531 |  |
| ASA-score 3&4 | 26 (44.1) | 26 (44.1) | 0 | 1 |  |
| Neoadjuvant chemotherapy | 54 (91.5) | 41 (69.5) | 0.58 | 0.006 |  |
| Previous abdominal surgery |  |  |  |  |  |
| Extrahepatic | 37 (62.7) | 23 (39.0) | 0.489 | 0.018 |  |
| Hepatic | 25 (42.4) | 23 (39.0) | 0.069 | 0.752 |  |
| *Procedural characteristics* | | | | |  |
| Time interval PVE to resection, days | 48 [36, 59] | 47 [36, 55] | 0.384 | 0.313 |  |
| Extent of resection |  |  | 0 | 1 |  |
| Right hepatectomy | 46 (78.0) | 46 (78.0) |  |  |  |
| Extended right hepatectomy | 13 (22.0) | 13 (22.0) |  |  |  |
| Part of two-stage hepatectomy | 28 (47.5) | 27 (45.8) | 0.03 | 1 |  |
| Major concurrent procedure(s) | 1 (1.7) | 2 (3.4) | 0.11 | 1 |  |
| *Disease characteristics* | | | | |  |
| Bilobar distribution* | 18 (30.5) | 24 (40.7) | 0.21 | 0.327 |  |
| Number of lesions | 5 [2.5, 6] | 4 [2, 8] | 0.15 | 0.651 |  |
| Size largest lesion, millimetres | 35 [20, 49.5] | 37.5 [18, 57.5] | 0.095 | 0.187 |  |
| Values are expressed in counts (percentages) or in median (IQR).  Abbreviations: SD, standardized difference; BMI, body mass index; ASA, American Society of Anaesthesiologists; PVE, portal vein embolization.  *At the second-stage in case of a two-stage hepatectomy | | | | |  |

| **Supplementary table 7.** Perioperative outcomes stratified by treatment group, after propensity score matching**, only high-volume centres** | | | |  |
| --- | --- | --- | --- | --- |
|  |  |  |  |  |
|  | Overall | | P |  |
|  |  |  |  |  |
|  | Laparoscopic | Open |  |  |
|  | n = 59 | n = 59 |  |  |
| *Intraoperative outcomes* | | | |  |
| Operative time, minutes | 330 [270, 392.3] | 240 [188, 300] | 0.004 |  |
| Estimated blood loss, millilitres | 600 [300, 1250] | 590 [332.5, 976] |  |  |
| Intraoperative PRBC transfusion | 14 (26.9) | 10 (22.7) | 1 |  |
| Number of PRBC transfused* | 2 [2, 3.5] | 2 [2, 2] | 0.750 |  |
| Pringle manoeuvre | 44 (74.6) | 30 (58.8) | 0.081 |  |
| Total Pringle time when used (minutes) | 69 [35, 100] | 30 [25, 44] | <0.001 |  |
| Conversion | 7 (11.9) |  |  |  |
| Bleeding | 4 (19.0) |  |  |  |
| Technical difficulty | 2 (9.5) |  |  |  |
| Oncological safety | 0 |  |  |  |
| Planned after mobilization | 1 (4.8) |  |  |  |
| Intraoperative unfavourable incidents |  |  | 0.824 |  |
| Grade I | 6 (11.5) | 6 (18.2) |  |  |
| Grade II | 3 (5.8) | 1 (3.0) |  |  |
| Grade III | 2 (3.8) | 0 |  |  |
| *Postoperative outcomes* | | | |  |
| Overall morbidity | 23 (39.0) | 27 (49.1) | 0.264 |  |
| Bile leak (≥ grade A) | 4 (6.8) | 5 (9.1) | 0.450 |  |
| Liver failure (≥ grade A) | 3 (5.2) | 5 (9.4) | 1 |  |
| Severe morbidity | 12 (20.3) | 8 (15.1) | 0.547 |  |
| Length of stay, days | 6 [4, 9] | 8 [7, 14] | <0.001 |  |
| Readmission | 7 (12.3) | 4 (10.8) | 1 |  |
| Radical resection margin (R0) | 42 (71.2) | 48 (84.2) | 0.190 |  |
| 90-day or in-hospital mortality | 3 (5.1) | 4 (6.8) | 1 |  |
| Values are expressed in counts (percentages) or in median (IQR).  Abbreviations: PRBC, packed red blood cell.  *For patients that received a transfusion | | | |  |

**Supplementary figure 1.** Overview of missing data

**
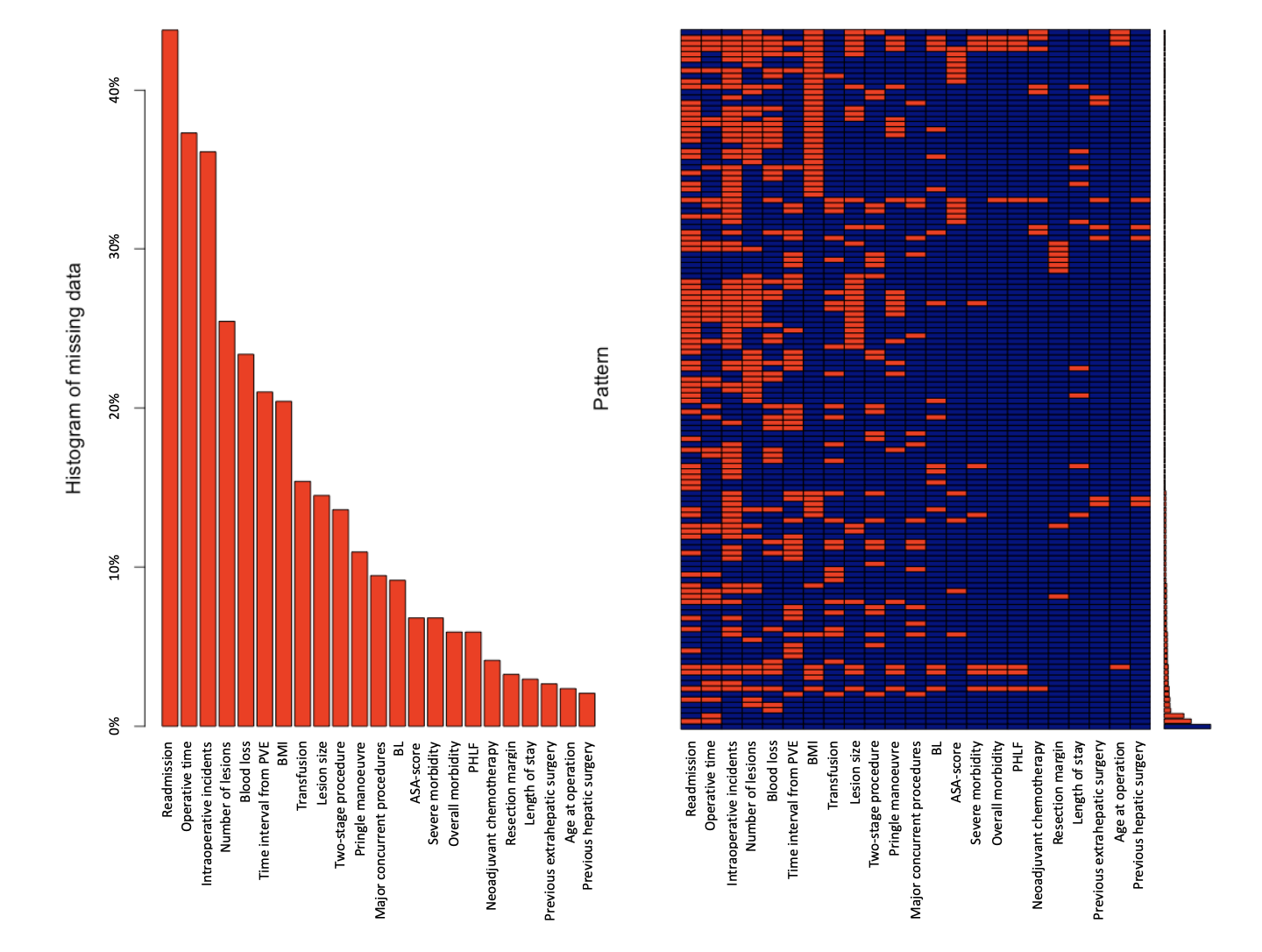
**
